# Antihypertensive Medication Use and Its Effects on Blood Pressure and Haemodynamics in a Tri-ethnic Population Cohort: Southall and Brent Revisited (SABRE)

**DOI:** 10.3389/fcvm.2021.795267

**Published:** 2022-01-14

**Authors:** Alun D. Hughes, Sophie V. Eastwood, Therese Tillin, Nish Chaturvedi

**Affiliations:** Medical Research Council (MRC) Unit for Lifelong Health & Ageing, Department of Population Science & Experimental Medicine, Institute of Cardiovascular Science, University College London, London, United Kingdom

**Keywords:** hypertension, anti-hypertensive agents, ethnicity, hemodynamic/drug effects, diabetes, hemodynamic, arterial compliance

## Abstract

**Objectives:**

We characterised differences in BP control and use of antihypertensive medications in European (EA), South Asian (SA) and African-Caribbean (AC) people with hypertension and investigated the potential role of type 2 diabetes (T2DM), reduced arterial compliance (C_a_), and antihypertensive medication use in any differences.

**Methods:**

Analysis was restricted to individuals with hypertension [age range 59–85 years; *N* = 852 (EA = 328, SA = 356, and AC =168)]. Questionnaires, anthropometry, BP measurements, echocardiography, and fasting blood assays were performed. BP control was classified according to UK guidelines operating at the time of the study. Data were analysed using generalised structural equation models, multivariable regression and treatment effect models.

**Results:**

SA and AC people were more likely to receive treatment for high BP and received a greater average number of antihypertensive agents, but despite this a smaller proportion of SA and AC achieved control of BP to target [age and sex adjusted odds ratio (95% confidence interval) = 0.52 (0.38, 0.72) and 0.64 (0.43, 0.96), respectively]. Differences in BP control were partially attenuated by controlling for the higher prevalence of T2DM and reduced C_a_ in SA and AC. There was little difference in choice of antihypertensive agent by ethnicity and no evidence that differences in efficacy of antihypertensive regimens contributed to ethnic differences in BP control.

**Conclusions:**

T2DM and more adverse arterial stiffness are important factors in the poorer BP control in SA and AC people. More effort is required to achieve better control of BP, particularly in UK ethnic minorities.

## Introduction

Elevated blood pressure (BP) is a major global public health problem and a leading preventable cause of cardiovascular morbidity and mortality. Levels of BP and prevalence of hypertension differ quite markedly between countries and between ethnic groups within populations for reasons that are poorly understood (1).

In the UK and Europe, BP and prevalence of hypertension has generally been found to be higher in people of African/African-Caribbean (AC) ethnicity compared with people of European ancestry (EA) (2), findings in South Asian (SA) people are less consistent (3, 4). BP control in people with hypertension is also poorer in people of African heritage (5, 6), with data in SA being more equivocal (4, 5).

The extent to which differences in efficacy of antihypertensive medication contribute to ethnic differences in BP control in hypertension remains uncertain. Differences in levels of BP control may reflect several factors, not only drug effectiveness. There is evidence that some BP-lowering agents, notably beta-blockers and inhibitors of the renin-angiotensin system (RAS) are comparatively less effective in people of African ancestry (7, 8), while calcium channel blockers (CCB) and diuretics may be more effective (7–9). Although subsequent data examining the achieved differences in BP in a ‘real world setting' suggests differences may be minimal (10). There is limited comparable data in SA, but what exists suggests little or no difference in efficacy of antihypertensive agents (11, 12). On the basis of evidence from trials and mechanistic considerations, the UK National Institute for Health and Care Excellence (NICE) in 2006 recommended that patients of Black African or Black Caribbean ethnicity, should receive either a calcium-channel blocker or a thiazide-type diuretic as first-line therapy (13).

Type 2 diabetes mellitus (T2DM) often accompanies high BP and people with T2DM have a ~2-fold increased risk of hypertension. Rates of T2DM are 3–4 fold higher, respectively, in people of AC and SA ancestry compared with EA in the UK, and this may contribute to differences in hypertension prevalence (14). The greater prevalence of T2DM may also influence the efficacy of antihypertensive medication, since smaller reductions in systolic BP in response to antihypertensive therapy have been observed in T2DM and more therapies may be required to achieve equivalent BP control in T2DM (15, 16). In contrast, current evidence suggests other common risk factors, such as sex, cholesterol and smoking have little or no influence on the response to antihypertensive treatment (15).

Elevated systolic BP and wider pulse pressure in older age is largely attributable to an increase in arterial stiffness (i.e., a reduction in arterial compliance, C_a_) (17), and increased arterial stiffness may account for the high rates of uncontrolled hypertension in diabetes (18). Previous studies have generally but not invariably found higher arterial stiffness (or lower C_a_) in older people of AC or SA ethnicity (19–21). These differences may be at least partially due to differences in prevalence ofT2DM and hyperglycaemia (19–21).

The aims of this study were to characterise ethnic differences in BP control and use of antihypertensive medications in people with hypertension from a multi-ethnic population sample of older people in the UK. We hypothesised that BP control would be poorer in ethnic minority groups and that this would be due to factors associated with ethnicity, principally, the greater prevalence T2DM and greater arterial stiffness making BP harder to control. We also investigated whether responses to major classes of antihypertensive agents differed by ethnicity.

## Methods

### Study Population

Southall and Brent Revisited (SABRE) is a UK population-based cohort of European Ancestry (EA), South Asian (SA), and African Caribbean (AC) adults. Details of the cohort have been published (22, 23). In brief, participants, aged 40–69 years, were recruited largely from primary care in West London, EA and SA recruits were predominantly male by design and baseline measurements were performed between 1988 and 1991 (22). Surviving participants (*N* = 3,433) were invited to attend the 20-year follow-up clinic for investigation between 2008 and 2011 and a total 1,438 participants attended. The current analysis was limited to all participants with hypertension (71% of total participants–EA 61%, SA 79%, AC 82%). Participants were excluded from the analysis if they had evidence of heart failure, atrial fibrillation or dysrhythmias, or invalid blood pressure data. A flow diagram of participant numbers and exclusions is shown in the Supplementary Figure S1. The study was approved by the St Mary's Research Ethics Committee, and all participants gave written informed consent.

### Investigations

Participants fasted and refrained from alcohol, smoking, and caffeine for ≥12 h before attendance and were asked not to take their medication on the morning of the clinic visit. A questionnaire was completed to obtain health behaviours, medical history, and medication. Height and weight were measured using a standardised protocol and body composition was measured using a Tanita BC 418 body composition analyzer. Seated brachial blood pressure was measured after 5–10 min rest using an automatic Omron 705IT according to ESH guidelines (24). An appropriately sized cuff was placed on to the left upper arm, 3 recordings were taken 2 min apart, and the second and third recordings were averaged. Blood pressure waveforms were acquired using a SphygmoCor device; measurements were acquired over at least six cardiac cycles and calibrated to brachial SBP and DBP according to the manufacturer's instructions. The commonest blood pressure targets in the UK for hypertension during this period were 140/90 or 130/80 mmHg in people with diabetes [reviewed in (25)], so achievement of BP control was therefore categorised on this basis. Since it was possible that differences in levels of BP control were explained by differences in prevalence of T2DM we also examined achievement of BP control in people without diabetes across the three ethnic groups.

Echocardiography was performed by two experienced cardiovascular physiologists using a Philips iE33 ultrasound machine. Data sets were acquired over 4 cardiac cycles during held respiration and analysed offline using Philips Qlab 7.0. Estimation of stroke volume, aortic flow velocity and left ventricular outflow tract diameter (LVOT) and calculation of left ventricular mass were performed according to American Society of Echocardiography (ASE) recommendations (26). Left ventricular mass was indexed to body surface area (LVMI) and left ventricular hypertrophy was categorised as >125 g/m^2^ for men and >110 g/m^2^ for women (27). Reproducibility of BP and echocardiography measures has been reported previously (28, 29).

Fasting blood samples were taken and stored at −80°C prior to analysis. Diabetes mellitus (T2DM) was defined according to the 1999 WHO guidelines (30), or physician diagnosis or receipt of anti-diabetes medications. Hypertension was defined as physician-diagnosed hypertension or participant-reported hypertension or receipt of BP-lowering medication. Study visit BP was not used to diagnose hypertension. Estimated glomerular filtration rate (eGFR) was calculated using the CKD Epi creatine/cystatin C method (31). Medications were based on participant self-report and general practitioner records. Smoking was classified into current or non-smoker based on self-report. Self-reported alcohol consumption was categorised according to UK guidelines into none, ≤ 14 units per week, or >14 units per week. Physical activity was summarised as the estimate of weekly energy expenditure in daily activities plus sport, walking, cycling, and strenuous activities.

Additional calculations were performed on BP waveforms using custom-written software in MATLAB (32). (https://github.com/adh30/Sphygmocor-Reservoir). Calculation of arterial compliance (C_a_) and systemic vascular resistance (SVR) was performed using the area method described by Liu et al. (33), since C_a_ and SVR are expected to be related to height, statistical models including C_a_ and SVR also included height.

### Statistics

All analyses were performed using Stata/MP 17.0 for Windows. Continuous sample data are summarised as means (SD) and categorical data as frequency (%). Comparison of sample characteristics by ethnicity was performed using analysis of variance for continuous variables followed by Tukey's test and categorical data were compared using a Chi^2^ test, followed by a logistic or ordered logistic regression for individual comparisons as appropriate. Comparisons between individual ethnic groups were made only if there was evidence against the null hypothesis from the omnibus test and Europeans were used as the reference group for all comparisons. Given the known differences in T2DM prevalence by ethnicity and the different BP targets in diabetes, stratified analyses by T2DM status were planned *a priori*. Global differences in antihypertensive medication use between ethnic groups were assessed by the normalised mutual information using the *dseg* user contributed program in Stata, which has previously been used to assess racial segregation and has advantages for non-dichotomous classes with multilevel structure (34).

Multivariable regression was performed using full-information maximum likelihood, or generalised structural equation modelling with equation-wise deletion, as appropriate, to minimise bias due to missingness under the assumption of missing at random. Choice of potential explanatory variables of differences in BP control was based on *a priori* knowledge (35). Treatment effects (TE) were estimated using augmented inverse probability weighting with conditioning on age, sex, T2DM, years of education, physical activity, % fat and alcohol consumption. % fat was included in model in preference to body mass index (BMI) since BMI is an inappropriate measure of adiposity across different ethnic groups (36). The TE method is a “doubly robust” method that models both the treatment and the outcome and is consistent even if one of the models is mis-specified (37). Potential outcomes and average treatment effects (ATE) were estimated using this approach. Results of regression models are presented as means [95% confidence intervals (95%CI)]. Inference was based on a combination of *p*-values, effect sizes and 95%CI, no adjustment was made for multiple comparisons.

## Results

Participants from the three ethnic groups were of similar age but there were slightly more males amongst the SAs than EA. By design there were more women in the sample of AC people (Table 1).

**Table 1:** Characteristics of participants by ethnicity.

**Variable**	**Ethnicity**									
	**EA**			**SA**			**AC**			
	***N***	**Mean/%**	**(SD)**	***N***	**Mean/%**	**(SD)**	***N***	**Mean/%**	**(SD)**	**χ^2^ and *F-*test *p*-values**
Age, years	328	70.4	(6.1)	356	69.4	(6.3)	168	70.5	(5.6)	0.051
Male sex	259	79.0%		303	85.1%		82	48.8%		<0.001
Peripheral systolic pressure, mmHg	317	145.0	(16.4)	335	144.1	(15.9)	166	145.5	(15.7)	0.605
Peripheral diastolic pressure, mmHg	317	85.6	(10.4)	335	83.7	(10.7)	166	86.9	(10.2)	0.003
Central systolic pressure, mmHg	317	135.1	(16.1)	335	135.0	(15.9)	166	136.6	(16.1)	0.547
Arterial compliance, ml/mmHg	292	1.7	(0.6)	311	1.5	(0.5)	152	1.5	(0.4)	<0.001
Systemic vascular resistance, mmHg/L	311	1.3	(0.4)	326	1.4	(0.4)	161	1.5	(0.4)	<0.001
Heart rate, bpm	317	64.8	(11.0)	335	62.9	(11.4)	166	63.4	(12.3)	0.095
Stroke index	321	41.5	(9.0)	347	42.4	(9.3)	163	39.5	(7.7)	0.003
Height, cm	328	170.2	(8.6)	356	166.0	(8.6)	168	165.0	(8.5)	<0.001
BMI, kg/m^2^	327	29.0	(4.9)	356	26.7	(3.9)	168	29.2	(5.3)	<0.001
Fat percent, bioimpedance	323	30.6	(7.8)	354	27.5	(7.3)	165	32.3	(8.8)	<0.001
HbA1c, mmol/mol	327	43.3	(8.0)	354	50.0	(13.4)	165	49.0	(12.8)	<0.001
eGFR, ml/min/1.73 m^2^	322	65.0	(16.6)	348	61.4	(16.3)	163	79.6	(20.7)	<0.001
Energy expenditure in physical activity, MJ/wk	310	9.7	(4.6)	344	9.2	(4.3)	152	8.8	(3.7)	0.081
Years of education	326	10.9	(2.5)	338	12.8	(3.7)	166	11.2	(2.9)	<0.001
Number of antihypertensive agents	328	2.1	(1.4)	356	2.7	(1.4)	168	2.6	(1.5)	<0.001
Receiving antihypertensive medication	290	88.4%		343	96.3%		157	93.5%		<0.001
BP controlled, *N* %	142	43.3%		102	28.7%		59	35.1%		<0.001
Diabetes mellitus, *N* %	84	25.6%		177	49.7%		77	45.8%		<0.001
Coronary heart disease, *N* %	84	25.6%		145	40.7%		23	13.7%		<0.001
Stroke, *N* %	16	4.9%		17	4.8%		18	10.7%		0.016
Left ventricular hypertrophy, *N* %	62	18.9%		47	13.2%		39	23.2%		0.012
**Alcohol consumption category**										<0.001
None	54	16.5%		45	12.6%		50	29.8%		
≤ 14 units per week	214	65.2%		279	78.4%		115	68.5%		
>14 units per week	60	18.3%		32	9.0%		3	1.8%		
Current smoker	18	7.7%		17	10.2%		7	7.7%		0.634

*AC, African Caribbean ethnicity; BP, blood pressure; eGFR, estimated glomerular filtration rate; EA, European ethnicity; LVH, left ventricular hypertrophy; SA South Asian ethnicity. Comparisons of groups were made using one-way analysis of variance*.

Compared with EA, SA, and AC were shorter; BMI was lower in SA; % fat was lower in SA and higher in AC; eGFR was lower in SA and higher in AC; T2DM was more prevalent in both SA and AC (and HbA1c was higher); years of education was higher in SA and alcohol consumption was lower in AC. Prevalence of CHD was higher in SA and lower in AC, while a history of stroke was commoner in AC and LVH was less frequent in SA. Smoking patterns and physical activity levels did not differ by ethnicity. Brachial and central systolic BP were similar by ethnicity, but diastolic BP and heart rate was lower in SA, while stroke index was slightly lower in AC.

### Antihypertensive Treatment and BP by Ethnicity

SA and AC people were more likely to receive treatment for high BP and received a greater average number of antihypertensive agents (Table 1), but despite this a smaller proportion of SA and AC achieved control of BP to target (Table 1). When, only people without T2DM were included in analyses, levels of control remained slightly poorer in ethnic minority individuals [number (%) with BP controlled in people without diabetes: EA 124 (51%), SA 75 (42%), AC 41 (45%)], although differences were substantially attenuated.

**Figure 1:**
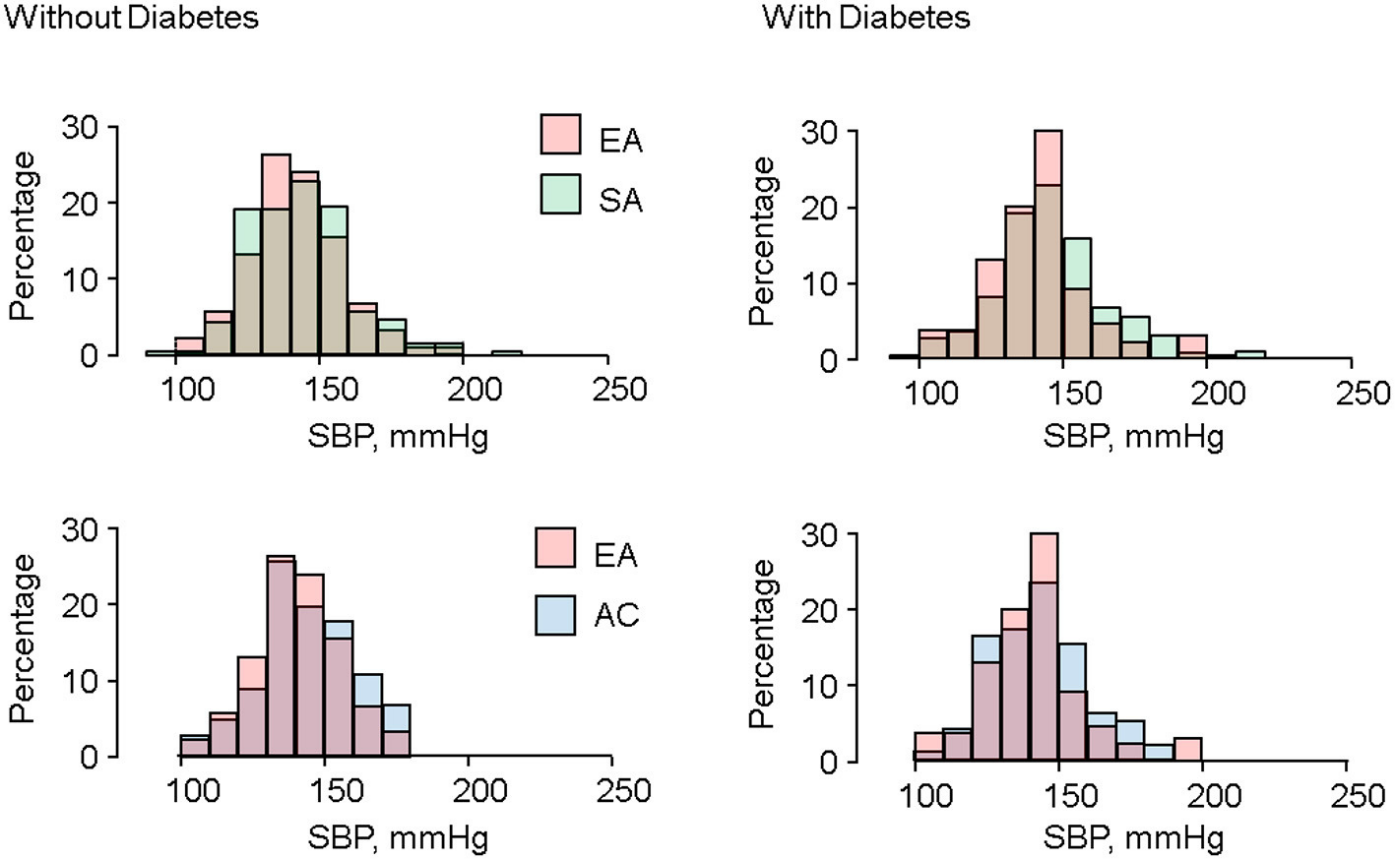
Ethnic differences in distributions of systolic BP in people with and without diabetes.

Distributions of systolic BP in SA and AC tended to be less peaked and more skewed to the right, particularly in the presence of T2DM (Figure 1). Distributions of cSBP and DBP are shown in Supplementary Figures 2, 3, respectively. Distributions of cSBP were similar to SBP, and there were no marked differences in distribution of DBP by ethnicity.

Adjustment for risk factors that might act as potential mediators of poorer control in ethnic minority groups (age, sex, T2DM, % fat, eGFR, LVH, physical activity, years of education, alcohol intake, prior CHD, or stroke) reduced, but did not abolish differences in the proportion of people with controlled BP (Table 2); most of the attenuation in this adjusted model was attributable to T2DM, which decreased the odds of achieving BP targets. However, this was not the sole explanation of differences as restricting the analysis to people without T2DM still resulted in unfavourable odds ratios (OR) of achieving BP control for both ethnic minorities, albeit less unfavourable than in the complete sample (Table 2).

**Table 2:** Blood pressure control in South Asian and African Caribbean people compared with Europeans with and without adjustment.

**Model**	**South Asian**		**African Caribbean**	
	**OR (95% CI)**	***P***	**OR (95% CI)**	***P***
Unadjusted	0.53 (0.38, 0.72)	<0.001	0.71 (0.48, 1.04)	0.08
Model 1	0.52 (0.38, 0.72)	<0.001	0.64 (0.43, 0.96)	0.03
Model 2	0.55 (0.36, 0.80)	0.002	0.75 (0.46, 1.21)	0.24
Model 3	0.70 (0.47, 1.04)	0.08	0.70 (0.43, 1.16)	0.17
Model 4	0.52 (0.35, 0.78)	0.001	0.74 (0.44, 1.23)	0.25
Model 5	0.65 (0.42, 1.00)	0.05	0.84 (0.49, 1.43)	0.52

*Odds ratios (OR) with 95% confidence intervals (95% CI) are shown for Model 1, age, sex; Model 2, Model 1 plus T2DM, % fat, eGFR, LVH, physical activity, years of education, alcohol intake, prior CHD or stroke; Model 3, Model 1 plus restricted to people without T2DM; Model 4 model 1 plus restricted to people receiving antihypertensive medication; Model 5, Model 2 plus arterial compliance*.

Restricting analysis to only people receiving antihypertensive treatment had little effect on estimates (Table 2). Additional adjustment for C_a_, which strongly increased the odds of achieving BP control (i.e., higher C_a_ was associated with greater probability of control), further attenuated differences (Table 2), although it did not completely abolish them. Addition of SVR to the model or substituting HbA1c for T2DM had minimal effects. A fully adjusted model results including haemodynamic variables is shown in Supplementary Table 1.

### Patterns of Antihypertensive Use by Ethnicity

**Figure 2:**
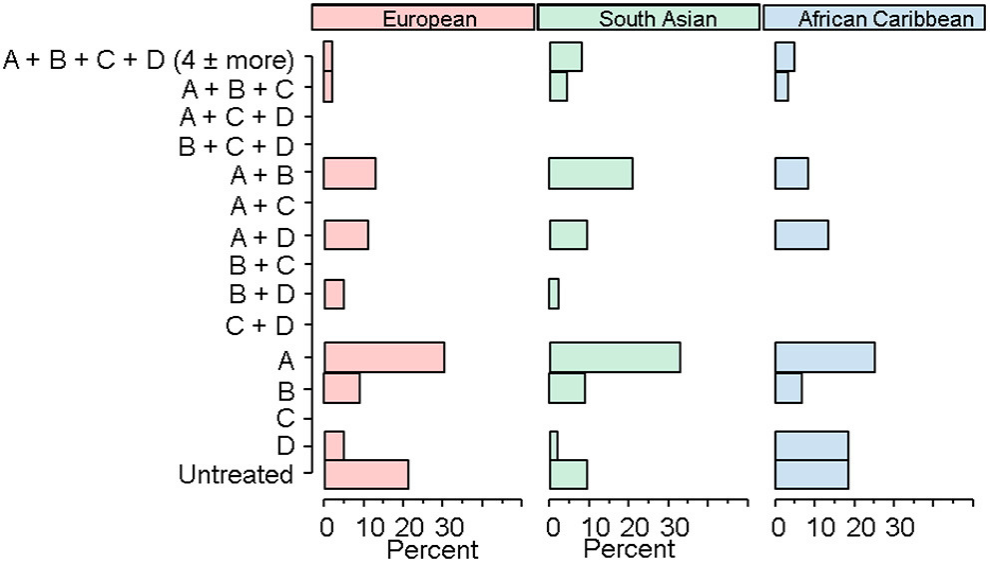
Ethnic patterns of antihypertensive medication.

Figure 2 shows the pattern of major classes and combinations of antihypertensive medication use by ethnicity. Overall patterns of antihypertensive therapy use appeared broadly similar by ethnicity, and the normalised mutual information was 0.051 indicative of very weak evidence of difference due to ethnicity. AC received more diuretic-based treatment regimens and both ethnic minority groups tended to receive more combination therapy, particularly 4 or more drugs (2%EA vs. 8%SA vs. 5%AC). A RAS inhibitor (A), either as monotherapy or in combination with another agent, was the most used therapy, with 59% of Europeans, 77% of SA, and 56% of AC receiving this class of antihypertensive agents. No participants in any ethnic group received a calcium channel blocker (C) as monotherapy, and no-one received the combinations of A + C, beta blocker (B) + C, C+ diuretic (D), B + C + D or A + C + D.

### Treatment Effects of Antihypertensives on Blood Pressure by Ethnicity

**Figure 3:**
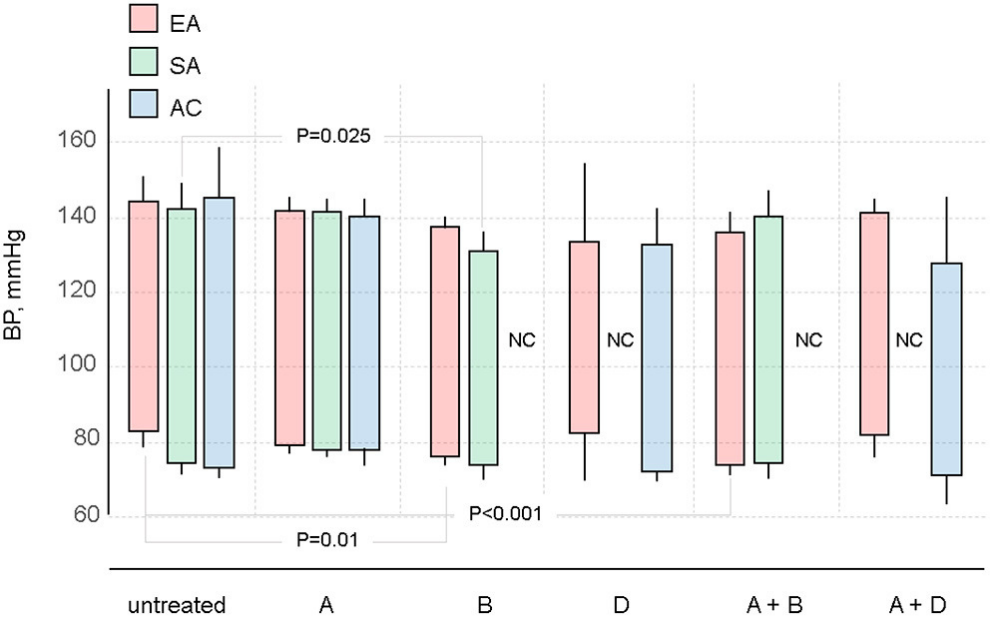
Potential outcome estimates for systolic and diastolic BP in different ethnic groups by antihypertensive treatment regimen. Abbreviations as in Table 3.

Treatment effects of the major classes of antihypertensives alone or in combination on BP, C_a_, and SVR were compared across ethnicities. Potential outcome systolic and diastolic BP are shown in Figure 3. Results for most of the treatments were broadly similar, although for many drugs or combinations, the estimates were too imprecise to draw firm conclusions; there was some evidence that B was more effective for lowering systolic BP in SA.

There was no convincing evidence that any of the evaluated regimens influenced C_a_ in any ethnic group (Table 3). There was some weak evidence that B reduced SVR in EA, but otherwise there was little evidence that any regimen affected SVR, although all estimates had wide confidence intervals.

**Table 3:** Treatment effects of antihypertensive regimens on arterial compliance and systemic vascular resistance by ethnicity.

	**EA**				**SA**				**AC**			
	**ATE**	**95% CI**		***p***	**ATE**	**95% CI**		***p***	**ATE**	**95% CI**		***p***
**Arterial compliance**												
A	0.08	(−0.20, 0.36)		0.57	−0.05	(−0.37, 0.26)		0.73	−0.01	(−0.20, 0.17)		0.88
B	0.34	(0.01, 0.68)		0.06	0.02	(−0.33, 0.36)		0.93	NC			
D	NC				NC				−0.03	(−0.36, 0.30)		0.85
A + B	0.05	(−0.26, 0.36)		0.76	−0.17	(−0.48, 0.14)		0.28	NC			
A + D	0.02	(−0.40, 0.44)		0.93	NC				NC		
	**PO**	**95% CI**			**PO**	**95% CI**			**PO**	**95% CI**		
Untreated	1.62	(1.38, 1.86)			1.63	(1.35, 1.91)			1.42	(1.29, 1.54)		
	**ATE**	**95% CI**		***p***	**ATE**	**95% CI**		***p***	**ATE**	**95% CI**		***p***
**Systemic vascular resistance**												
A	−0.11	−0.26	0.03	0.14	0.03	−0.10	0.16	0.67	−0.07	−0.41	0.28	0.70
B	−0.20	−0.37	−0.04	0.01	0.07	−0.09	0.22	0.38	NC			
D	NC				NC				0.09	−0.34	0.51	0.69
A + B	−0.09	−0.26	0.07	0.27	0.25	−0.05	0.56	0.1	NC			
A + D	−0.06	−0.30	0.18	0.64	NC				NC			
	**PO**	**95% CI**			**PO**	**95% CI**			**PO**	**95% CI**		
Untreated	1.39	1.28	1.50		1.39	1.29	1.49		1.57	1.25	1.89	

*A, renin-angiotensin system blocker; B, beta-blocker; C, calcium channel blocker; D, diuretic; NC, not calculated due to small numbers; ATE, average treatment effect; PO, potential outcome*.

## Discussion

Older people with hypertension from a UK tri-ethnic population-based sample in the UK had universally poor BP control. While average systolic pressures did not differ by ethnicity, attainment of BP targets in both SA and AC people was worse than in EA individuals, despite people in minority ethnic groups receiving more antihypertensive agents on average. Much, but not all, of this difference in BP control may be attributable to a higher burden of T2DM in the minority ethnic groups, but lower C_a_ (higher arterial stiffness) may also make an important contribution. Higher SVR was, if at all, associated with better likelihood of achievement of target BP.

At the time of the study contemporary guidelines in the UK required lower targets to be achieved in T2DM (38); this is likely to have contributed to the poorer levels of control as lower targets will naturally be more difficult to achieve, but there is also evidence that T2DM may reduce the efficacy of antihypertensive agents (15, 16). Antihypertensive agents can reduce arterial stiffness (39), but much of this effect may depend on BP reduction, as the elastic behaviour of arteries shows a non-linear incremental relationship with BP (40), and evidence for a direct effect of antihypertensive agents on intrinsic stiffness of arteries is limited (39, 40). Poorer arterial stiffness has previously been reported in these ethnic groups (19–21). Reduced C_a_ in SA and AC may be linked to the greater prevalence of T2DM or dysglycaemia in these ethnic groups, particularly since there is evidence that SA and AC experience a greater chronic glycaemic burden from childhood onwards (41), as well as having an earlier of onset T2DM (42, 43). Reasons for these ethnic differences in glycaemic burden, T2DM and C_a_ remain to be fully established, but there is evidence that obesity (particularly truncal obesity), low physical activity, psychosocial variables (e.g., racism) and socioeconomic disadvantage make important contributions (42, 44, 45).

Our observations are in keeping with earlier studies in UK which have found poorer rates of BP control in ethnic minorities (4–6). By design, SABRE recruited fewer women in the EA and SA samples which is a limitation (22). It should also be noted that the majority of SA recruits to SABRE were of Punjabi Sikh origin (46) and while migrants from the Indian subcontinent are generally at increased risk of T2DM compared with Europeans, the prevalence of T2DM varies between South Asian subgroups (47); so this may limit the generalizability of our findings. Our findings are consistent with a more recent European-wide study that found that African origin people were less likely to have their BP controlled, despite greater awareness of hypertension (4). We found little evidence that poorer control of BP in ethnic minorities in UK was attributable to marked differences in efficacy of antihypertensive regimens by ethnicity. This conclusion is in accord with some previous studies (10, 12); however, it should be stressed this part of our study had limited power and is vulnerable to uncontrolled confounding and bias by indication, consequently these findings should be viewed with considerable caution. We also note that BP was measured at trough in keeping with standard practise (48), but this will tend to under-estimate BP lowering for short-acting antihypertensive agents. Another limitation of our study is that there was a high rate of attrition in the overall sample and while there were no major differences in measured variables between baseline and clinic follow-up (22), this could have introduced bias. Previous data from randomised clinical trials (7–9) have indicated small differences in response to antihypertensive agents between people of European ancestry and African ancestry, but it is not clear how important these differences in efficacy are at a population level (10).

It is possible that therapeutic inertia on the physician's part or poor adherence on the patient's part contributed to the differences in BP control we observed, but our study lacks information on these questions. It was notable, however that utilisation of antihypertensive agents differed little by ethnicity and that patterns of utilisation seemed relatively uninfluenced by UK guidelines operating at the time of the study which made recommendations regarding preferred antihypertensive agents in ethnic minorities (38). This is particularly evident for calcium channel blockers which at the time of the study were advocated as first line agents for people of African or Caribbean ethnicity but were only used infrequently and not at all as monotherapy in any ethnic group. More recent data from more than 40,000 Medicare beneficiaries in the US are also consistent with the observation of poor physician concordance with guidelines (49).

Our observations of poorer BP control in ethnic minorities in the UK are important since both groups are at elevated risk of CVD: CHD and stroke for SA, and stroke for AC. While the issue of lower targets in T2DM remains under debate (50), our findings add to recent data suggesting that more aggressive antihypertensive treatment to achieve lower BP targets may be warranted (51). However, the poor levels of control reported in this study also underscore the difficulty of achieving such lower targets in a real-world setting. We speculate that initiation of antihypertensive therapy via a combination polypill, which has been reported to achieve better levels of BP control (52) might be a better strategy.

## Conclusion

In a tri-ethic population-based sample of older people control of hypertension was generally poor, but worse in people of AC and SA ethnicity. This poorer BP control occurred despite greater use of antihypertensive agents. Poorer BP control was partially accounted for by statistical adjustment for the greater prevalence of T2DM and more adverse arterial stiffness in these groups. Lowering blood pressure remains an important public health goal, and more effort is required to achieve better control of BP, particularly in ethnic minorities in UK.

## Data Availability Statement

Publicly available datasets were analysed in this study. This data can be found at: https://www.sabrestudy.org/home-2/data-sharing/.

## Ethics Statement

The studies involving human participants were reviewed and approved by St Mary's Research Ethics Committee. The participants provided their written informed consent to participate in this study.

## Author Contributions

AH contributed to conception and design of the study, performed the statistical analysis, and wrote the first draft of the manuscript. TT and AH contributed to data curation. NC and AH contributed to supervision and funding acquisition. SE, TT, and NC contributed to sections of the manuscript. All authors contributed to manuscript revision, read, and approved the submitted version.

## Funding

The SABRE study was funded at baseline by the Medical Research Council, Diabetes UK, and the British Heart Foundation. At follow-up the study was funded by the Wellcome Trust (067100, 37055891, and 086676/7/08/Z), the British Heart Foundation (PG/06/145, PG/08/103/26133, PG/12/29/29497, and CS/13/1/30327), and Diabetes UK (13/0004774). AH receives support from the British Heart Foundation, the Horizon 2020 Framework Programme of the European Union, the National Institute for Health Research University College London Hospitals Biomedical Research Centre, the UK Medical Research Council, the Wellcome Trust, and works in a unit that receives support from the UK Medical Research Council. SE was funded by a Diabetes UK Sir George Alberti Research Training Fellowship (Grant No. 17/0005588, https://www.diabetes.org.uk/).

## Conflict of Interest

NC receives funding from AstraZeneca to serve on data safety and monitoring committees for clinical trials. The remaining authors declare that the research was conducted in the absence of any commercial or financial relationships that could be construed as a potential conflict of interest.

## Publisher's Note

All claims expressed in this article are solely those of the authors and do not necessarily represent those of their affiliated organizations, or those of the publisher, the editors and the reviewers. Any product that may be evaluated in this article, or claim that may be made by its manufacturer, is not guaranteed or endorsed by the publisher.
