# Supplementary material for: Antihypertensive Medication Use and Its Effects on Blood Pressure and Haemodynamics in a Tri-ethnic Population Cohort: Southall and Brent Revisited (SABRE)
Source: Front Cardiovasc Med. 2022 Jan 14;8:795267. doi: 10.3389/fcvm.2021.795267 (PMC8795362; doi:10.3389/fcvm.2021.795267)
Supplement: Supplementary file 1 [file Data_Sheet_1.docx]

# Supplementary data

| **Variable** | **Odds ratio** | **95% CI** | | **P** | **Standardized Odds ratio** |
| --- | --- | --- | --- | --- | --- |
| Age | 0.99 | 0.95 | 1.02 | 0.46 | 0.92 |
| Female sex | 2.46 | 1.29 | 4.69 | 0.01 | 1.45 |
| South Asian | 0.65 | 0.42 | 0.99 | 0.05 | 0.81 |
| African Caribbean | 0.81 | 0.47 | 1.37 | 0.43 | 0.92 |
| T2DM | 0.30 | 0.20 | 0.45 | <0.001 | 0.56 |
| % fat | 0.99 | 0.96 | 1.02 | 0.65 | 0.95 |
| eGFR | 1.00 | 0.99 | 1.02 | 0.67 | 1.05 |
| LVH | 0.40 | 0.23 | 0.68 | <0.001 | 0.72 |
| Physical activity | 0.99 | 0.95 | 1.03 | 0.55 | 0.95 |
| Years Education | 1.04 | 0.98 | 1.10 | 0.22 | 1.12 |
| Alcohol | 0.71 | 0.50 | 1.00 | 0.05 | 0.83 |
| CHD | 1.44 | 0.97 | 2.14 | 0.07 | 1.18 |
| Stroke | 0.83 | 0.38 | 1.81 | 0.64 | 0.96 |
| C_a_ | 3.16 | 2.07 | 4.82 | <0.001 | 1.87 |
| SVR | 1.47 | 0.81 | 2.69 | 0.21 | 1.15 |

**Table S1.** Multivariable association between exposures and odds ratio and standardized odds ratio for blood pressure control. Odds ratios were calculated using a generalized structural equation model with a logit link and each standardized to the standard deviation of the independent variable. Abbreviations: C_a_, arterial compliance; CHD, coronary heart disease; eGFR, estimated glomerular filtration rate; LVH, left ventricular hypertrophy; SVR, systemic vascular resistance; T2DM, type 2 diabetes mellitus.


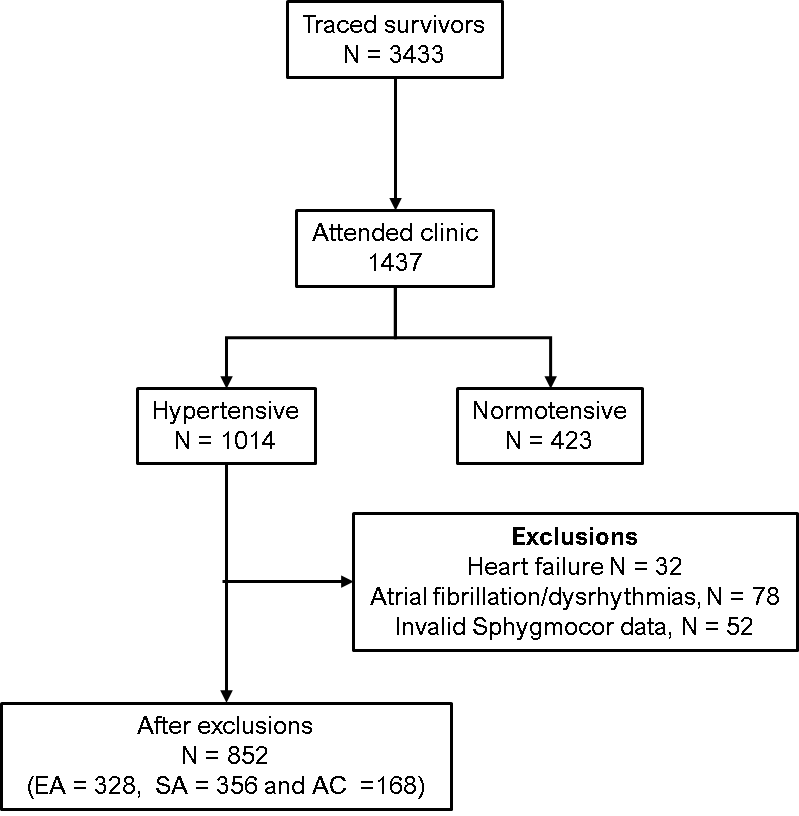


**Figure S1.** Participant numbers following application of inclusion and exclusion criteria.

**Figure S2.** Ethnic differences in distributions of central systolic pressure in people with and without diabetes.

**Figure S3.** Ethnic differences in distributions of diastolic pressure (DBP) in people with and without diabetes.
